# The Down Syndrome-Associated Protein, Regulator of Calcineurin-1, is Altered in Alzheimer’s Disease and Dementia with Lewy Bodies

**DOI:** 10.4172/2161-0460.1000462

**Published:** 2019-03-06

**Authors:** Malakooti N, AIBL Research Group, Fowler C, Volitakis I, McLean CA, Kim RC, Bush AI, Rembach A, Pritchard MA, Finkelstein DI, Adlard PA

**Affiliations:** 1 Florey Institute of Neuroscience and Mental Health, University of Melbourne, Parkville, 3010, Victoria, Australia; 2 The Australian Imaging, Biomarkers & Lifestyle Flagship Study of Ageing, Australia; 3 Department of Pathology and Laboratory Medicine, University of California, Irvine, USA; 4 Department of Biochemistry and Molecular Biology, Monash University, Clayton, 3168, Victoria, Australia

**Keywords:** Alzheimer’s disease, Down syndrome, Dementia

## Abstract

There is a known relationship between Alzheimer’s disease (AD) and Down syndrome (DS), with the latter typically developing AD-like neuropathology in mid-life. In order to further understand this relationship we examined intersectin-1 (ITSN1) and the regulator of calcineurin-1 (RCAN1), proteins involved in endosomal and lysosomal trafficking that are over-expressed in DS. We examined RCAN1 and ITSN1 levels (both long (-L) and short (-S) isoforms) and the level of endogenous metals in White Blood Cells (WBCs) collected from AD patients who were enrolled in the Australian Imaging, Biomarker and Lifestyle Study on Ageing (AIBL). We also examined RCAN1 and ITSN1-S and -L in post-mortem brain tissue in a separate cohort of patients with AD or other types of dementia including Dementia with Lewy Bodies (DLB) and non-Alzheimer’s disease dementia. We found that RCAN1 was significantly elevated in AD and DLB brain compared with controls, but there was no difference in the level of RCAN1 in WBCs of AD patients. There were no differences in the levels of ITSN1-L and −S between AD and the control, nor between other types of dementia and the control. We found that there were no differences in the levels of metals between AD and the control WBCs. In conclusion, our data demonstrate that RCAN1 is differentially regulated between the peripheral and central compartments in AD and should be further investigated to understand its potential role in dementia of AD and DLB.

## Introduction

The current worldwide incidence of dementia is believed to be fifty million people, and this number is expected to reach 75 million in 2030 if no cure is found (Alzheimer’s disease international, 2017) [1]. Alzheimer’s disease, the most common form of dementia, is characterised by neuropathological changes (including the development of β-amyloid plaques (Aβ), Neurofibrillary Tangles (NFTs) and other anatomical features that spread throughout the brain) that result in a variety of clinical symptoms including short-term and long-term memory loss, confusion, depression and language problems. Ultimately, patients can become severely demented, lose ambulation and are reduced to a behavioural repertoire consisting of a few basic reflexes [2,3]. The individual neuropathological features of the AD brain are not unique to this disease, and are found across a spectrum of disorders and species [4]. One such example is Down Syndrome (DS), where individuals develop Aβ neuritic plaques, tau-containing NFTs [5], Basal Forebrain Cholinergic Neurodegeneration (BFCN) and enlarged early endosomes. These features may be the result of an over-expression of multiple genes or an alteration in key proteins or discrete cellular pathways. It has also been suggested, however, that another shared pathology, enlarged early endosomes, may contribute to pathological processes in both AD and DS and may be mediated through common pathways. Two genes involved in endocytosis, both located on chromosome 21, are intersectin-1 (ITSN1) and the regulator of calcineurin-1 (RCAN1, formerly called Down syndrome candidate region 1) [6–9]. This is relevant because DS is characterised by a triplication of chromosome 21, which is also the location of the Amyloid Precursor Protein (APP) gene that ultimately gives rise to the Aβ protein that forms the plaques found in AD and DS.

Through endocytosis, neurons achieve the rapid vesicle recycling necessary for maintaining neurotransmission but endocytosis is also the process used by neurons and other cell types to take up macromolecules from the extracellular environment. Early endosomes receive extracellular molecules from the cell surface via fusion with clathrin-coated vesicles. Disrupted endocytosis has been postulated to result in abnormal uptake and trafficking through signalling endosomes of vital plasma membrane proteins, growth factors and receptors [10]. Early endosomes are the sites of internalisation of APP and apolipoprotein E, as well as the site of Aβ peptide generation, all of which contribute to the manifestation of AD [11]. In addition, defective neuronal growth factor signaling due to disturbances in endocytosis could be an early event in the manifestation of AD [12] which can lead to the formation of amyloid plaques, hyperphosphorylated tau and NFTs and BFCN [13]. In DS, enlarged endosomes are seen as early as 28 weeks of gestation in neurons [10], which precedes diffuse Aβ plaque deposition which appears at around 12 years of age, and is followed by mature Aβ plaques when the individuals are in their 30s [14]. In 2008 the genes associated with cognitive decline in the brains of aging individuals with AD were identified by profiling RNA expression of the whole genome in the frontal cortex and comparing them to their matched controls. Of relevance to this study, amongst the RNA transcripts significantly up-regulated in AD was the short isoform of intersectin-1 (ITSN1-S) [15]. Although this study reported that ITSN1 is overexpressed in the AD brain [15], there is nothing in the literature about the expression of ITSN1 at the protein level or in other types of dementia. It is of note, however, that ITSN1 protein levels have been examined in DS individuals that had concomitant AD pathology [16]. This study showed that DS individuals with AD pathology have a higher level of expression of ITSN1 in their frontal cortex compared with healthy controls, but interestingly DS individuals with diagnosis of AD had lower levels of both ITSN1-S and -L compared to DS without an AD diagnosis [16]. Similarly, previous studies have shown that the level of RCAN1 mRNA was increased in the AD brain [17,18] and that the level of RCAN1 protein was increased in the pyramidal neurons of the AD temporal lobe [19], but there is almost nothing in the literature about the levels of RCAN1 in other types of dementia. Ermak et al. [17] also showed that amyloid β1–42 stimulates production of RCAN1 mRNA in a cell culture model. Furthermore, Lloret et al. [20] showed that in a primary rat neuronal cell culture model in the presence of amyloid β, tau phosphorylation increased but silencing RCAN1 in these neurons blocked the hyperphosphorylation of tau indicating that RCAN1 has a role in tau phosphorylation.

For these reasons, we hypothesised that protein levels of both ITSN1 and regulator of calcineurin-1 RCAN1 would be altered in AD brain tissues, and may also be altered in related conditions, including Dementia with Lewy bodies (DLB) and non-Alzheimer’s disease dementia (non-AD; including corticobasal degeneration and supranuclear palsy). Changes in these proteins may implicate endocytic and lysosomal trafficking deficits across a broad suite of neurodegenerative diseases.

As our targets of interest have been found in various tissues throughout the body [21], we were also interested in determining whether or not they were changed in the periphery, as this might represent a potential biomarker [22,23] or provide some insight into the pathogenesis of disease. For these analyses we were fortunate to have access to white blood cells, which are a critical component of the peripheral compartment and may be involved either directly or indirectly in the pathogenesis of a variety of conditions including AD [24,25], from both healthy controls and AD patients from the Australian imaging, Biomarker and Lifestyle Study of Ageing (AIBL). Furthermore, both ITSN1 and RCAN1 are involved in transporting material inside and outside the cell; RCAN1 regulates vesicle exocytosis [26] and ITSN1-L isoform regulates the amount of secretory vesicle exocytosis and synaptic vesicle endocytosis [9]. Therefore, it is possible that they would be involved in the transportation of metals, which are reported to be involved in the pathogenesis of a variety of neurodegenerative diseases (such as AD). ITSN1 is also involved in receptor-mediated endocytosis, and it is reported to impact the internalization of the transferrin receptor, which is crucially involved in cellular iron regulation [27]. Hence, we hypothesised that if the level of ITSN1 and RCAN1 were altered in the periphery, then this may also translate to a change in metal levels. Hence, we assessed both protein (ITSN1 and RCAN1) and metal (including major elements such as copper, zinc, iron and calcium) content in the white blood cells.

## Materials and Methods

### Human brain tissue

Human post-mortem brain tissues used in this project were provided by the Victorian Brain Bank Network and the University of California Alzheimer’s Disease Research Center (UCI-ADRC) and the Institute for Memory Impairments and Neurological Disorders. The demographics of these cases are listed in Table 1. Samples included frontal and temporal cortices from controls with no neurological disorders (average ± SD; 80.8 ± 14.5 years of age), AD (78.3 ± 9.1 years of age), non-AD (including corticobasal degeneration and progressive supranuclear palsy cases; 77.7 ± 6.9 years of age), and DLB (82.6 ± 7.3 years of age) patients. There was no statistical difference in the average age of each of these cohorts. The average Post Mortem Interval (PMI) for the tissues were as follows; controls (22.1 ± 27.7 hours), AD (33.4 ± 22 hours), non-AD (28.6 ± 16.3 hours) and DLB (41.8 ± 22.7 hours). The variation in PMI is similarly large across all the groups, and there was no statistically significant difference between groups. The tissues were stored at −80°C until required.

### Human blood samples

White blood cell samples from fasting AD (n=50, 77.5±11.5 years of age) and age-matched healthy controls (n=20, 79 ± 10 years of age) were obtained from AIBL.

### Western blotting of human samples

Western blotting was used to quantify the relative levels of RCAN1, ITSN1 long and ITSN1 short isoforms in both human brain and white blood cell samples. Specific cohort sizes are shown in the figures. Post-mortem tissue was weighed and homogenized in 4× the volume in Phosphate Buffered Saline (PBS) containing 0.1% SDS and 0.1% Triton-100, supplemented with proteinase inhibitor tablets (Roche) and phosphatase inhibitors (Roche, Mannheim, Germany). White blood cell samples were homogenised in dH_2_O containing 0.1% SDS and 0.1% Triton-100, supplemented with proteinase inhibitor tablets (Roche) and phosphatase inhibitors (Roche, Mannheim,Germany). Each sample was sonicated for 10 cycles of 10 seconds on and 10 seconds off. Further, the samples were spun for 10 mins and the soluble phase collected for experiment. Protein concentrations of all the samples were initially quantified using a bicinchoninic (BCA) protein assay kit (Pierce, Thermo scientific, Rockford, USA) so that equal protein concentrations (40 μg) of each homogenised sample could be loaded per lane and subsequently resolved on 3–8% Criterion XT Tris-Acetate pre-cast gels (Bio-Rad, Hercules, CA, USA) using XT Tricine running buffer (Bio-Rad, Hercules, CA, USA). This was followed by electroblotting onto polyvinylidene fluoride (PVDF) membranes (Immobilon-P) using transfer buffer containing 5% methanol.

Membranes were incubated in milk (5%w/v) followed by applying the primary rabbit anti-ITSNl (1:750) (Abcam, Cambridge, UK) in blocking buffer (5% w/v fat-free milk in TBS containing 0.1% Tween-20, pH 8.0) and anti RCAN-1 (1:1000; MorphoSys AG, Planegg, Germany) in signal boost solution 1 buffer (Calbiochem, Darmstadt, Germany) and incubated overnight at 4°C.

Immunoreactive proteins were detected using HRP-conjugated rabbit anti-mouse (1:2000; Dako, Glostrup, Denmark) in blocking buffer (5% w/v fat-free milk in TBS containing 0.1% Tween-20, pH 8.0) or HRP-conjugated goat anti human (1:2000, Jackson ImmunoResearch Laboratories, West Baltimore, USA) in signal boost solution buffer 2, respectively. ITSN1 membranes were incubated with Amersham ECL western blotting detection reagent and RCAN1 was visualized using Luminata Forte western HRP substrate (Millipore, Burlington, USA). Images of the western blots were taken using an ImageReader LAS-3000 (FujiFilm, Tokyo, Japan) and the abundance of proteins quantified (density of bands at the given molecular weight) using ImageQuant software (GE Healthcare, Fairfield, USA). The data are then presented as relative band intensity.

### Metal analysis by inductively coupled plasma mass spectrometry

Due to our long standing interest in the role of metals in the pathogenesis of AD, we assessed metal levels in the white blood cells from both AD and healthy control patients. Whilst this in of itself is interesting, to determine whether there was any potential involvement of ITSN1 and RCAN1 in metal homeostasis in the periphery (which may reflect central metal changes), then we correlated the protein and metal levels. We utilized Inductively Coupled Plasma Mass Spectrometry (ICPMS) as described previously [28]. Triplicates of each sample were measured.

### Statistical analysis

Data for human tissue are presented as box and whiskers for comparison of AD and controls, and were compared using a two-tailed student’s t-test. Unless otherwise stated, data are presented as means ± SEM for other types of dementia and controls, and were compared by one way ANOVA with Dunnet’s post-hoc test. Data for WBCs metals were analysed using a two-tailed student’s t-test. A Pearson’s two-tailed correlation test was used to test the correlation between proteins and metals. All statistical analyses were performed using GraphPad Prism 6 software (GraphPad Software, La Jolla, CA, USA). On all the figures, the significance is denoted by the following: *p<0.05, **p<0.01 and ***p<0.001.

## Results

### Intersectin-1 protein levels are unchanged in brain tissue in various neurological diseases

**Figure 1:**
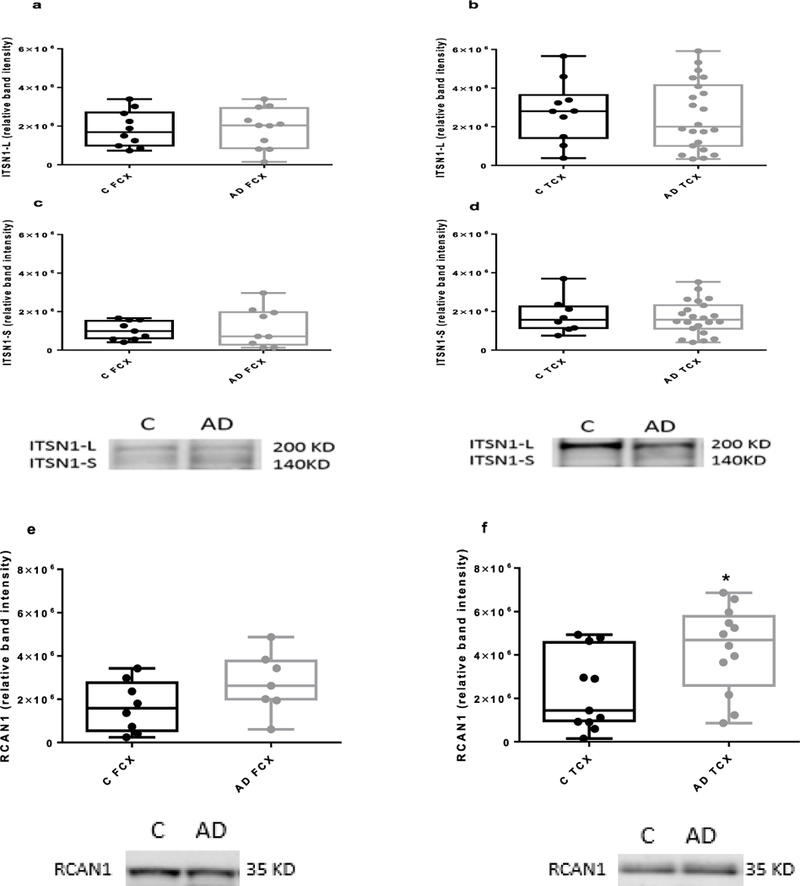
Levels of ITSN1 and RCAN1 proteins in frontal and temporal cortices of AD patients. Western blotting was performed on homogenates of human post-mortem brains (n=6– 11 frontal cortex (FCX), n=11–22 temporal cortex (TCX)) and age-matched controls (C; n=6– 10 FCX, n=8–10 TCX). Plots (a, b, c, d) show levels (densitometry) of ITSN1-S and L in post- mortem brains of AD patients compared with age-matched controls e) There is no difference between the AD and control in levels of RCAN1 frontal cortex. f) The level of RCAN1 protein is significantly higher in the AD temporal cortex compared with the control. Representative blots are shown. The box and whisker graphs show median, minimum and maximum values. An unpaired two-tailed t-test was used for statistical analysis (p=0.022). *p<0.05.

**Figure 2:**
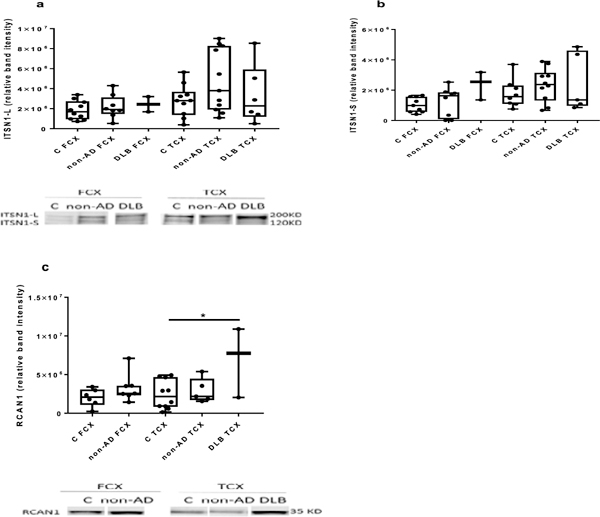
ITSN1 and RCAN1 protein levels in different types of dementia. There were no differences in the level of ITSN1 in different types of dementia compared with the controls (a,b)[(for frontal cortex (FCX): n=10 for control (C), n=8 for non-AD, n=3 for DLB; and for temporal cortex (TCX): n=10 for control, n=11 for non-AD, n=6 for DLB]. However, the level of RCAN1 is elevated in DLB temporal cortex compared with control (c) (for frontal cortex: n=9 control and n=8 for non-AD (due to tissue limitations and technical difficulties, we only had an n=1 for the frontal cortex from DLB patients, and so left this out of the analysis); for temporal cortex: n=10 control, n= 6 for non-AD, n= 3 for DLB; p= 0.011 for DLB). The representative blots of ITSN1 and RCAN1 protein levels in different types of dementia are shown (the “split” lanes are from non-adjacent samples on the blot). The box and whisker graphs show median, minimum and maximum values. One way ANOVA with Dunnett’s post-hoc test. The RCAN1 measurements for DLB FCX cases (in Panel 2c) were not included due to insufficient numbers. *p<0.05.

There were no differences between the levels of ITSN1 proteins in frontal and temporal cortices of AD patients compared with the controls (unpaired two-tailed t-test; n=6–10 for control frontal cortex and n=6–12 for AD frontal cortex; n =8–10 control temporal cortex, n=11–22 for AD temporal cortex (Figure 1a–1d). Similarly, there were no differences between other types of dementia and the controls (one way ANOVA with Dunnet’s post-hoc test; Figure 2a and Figure 2b).

### RCAN1 levels are elevated in the temporal cortex in AD and DLB

Assessment of RCAN1 levels in frontal and temporal cortices from our cohort of neurological diseases (Figure 1e, Figure 1f and Figure 2c) revealed a significant elevation only in the temporal cortex of both AD and DLB tissue, as compared to controls (Figure 1f and Figure 2c). There were no other differences noted.

### Intersectin-1 and RCAN1 protein levels in white blood cells in AD

**Figure 3:**
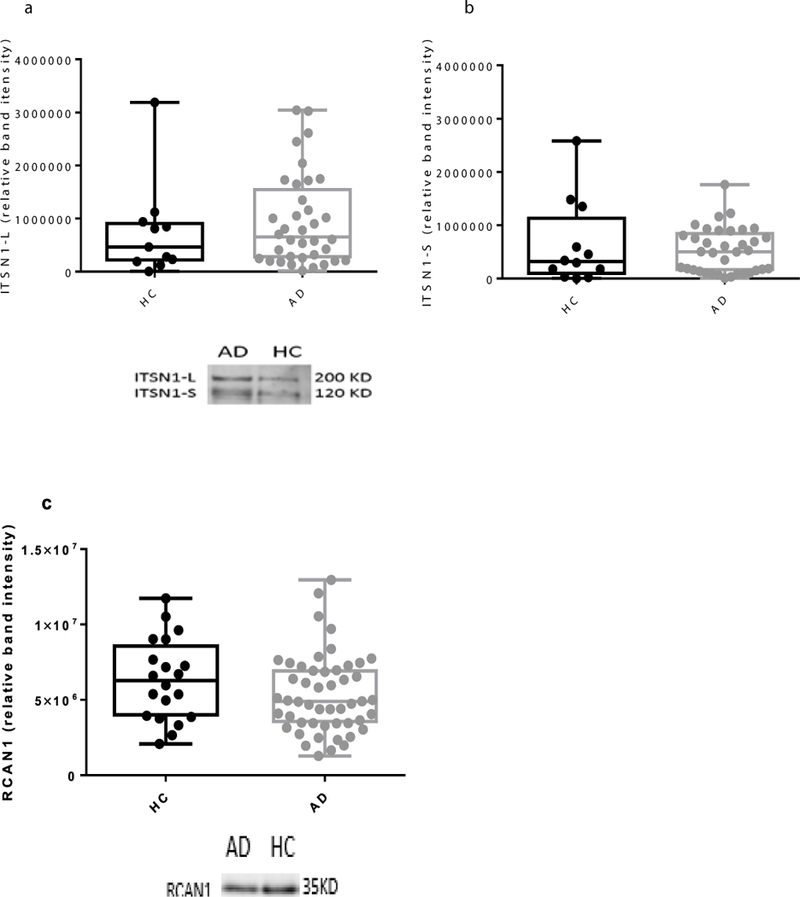
Levels of ITSN1-L, ITSN1-S and RCAN1 in AD white blood cells. Western blotting was performed on homogenates of white blood cells of AD (n=36–50) and HC(n=11– 20). a, b and c) There were no differences between the groups for the level of ITSN1-L, ITSN1- S and RCAN1. Representative blots are shown. The box and whisker graphs show median, minimum and maximum values.

The protein levels of both the short and long isoforms ofITSN1 (Figure 3a and Figure 3b), as well as RCAN1 (Figure 3c) were no different between the AD and healthy controls.

### Metal analysis in white blood cells, and correlation with RCAN1 and ITSN1

There were no differences in the levels of any metals measured (iron, zinc, copper, calcium, magnesium, manganese, aluminium, lead or selenium) between the AD and the control white blood cells (Table 2). We also examined whether there was a correlation between the level of the proteins of interest (RCAN1 or ITSN1) and the level of each metal across our two cohorts using a Pearson’s two-tailed correlation. There was no correlation between the levels of any of the proteins and metals (Table 2).

## Discussion

This study has demonstrated that there are elevated levels of RCAN1 protein in both AD and DLB temporal cortices, as compared to matched controls. These findings are consistent with a previous study conducted in AD patients, where it was shown that RCAN1 mRNA was elevated (~ two fold) in the cerebral cortex (areas A10 and A22) and hippocampus, but not the cerebellum, of AD patients [17]. This study also demonstrated an association with NFTs, such that RCAN1 mRNA was significantly higher in patients with extensive NFTs (~ three fold). In a cell culture system, it was also shown that amyloid β1–42 increases RCAN1 mRNA [17]. This current study, therefore, extends on the previous work that only examined changes in RCAN1 mRNA, to demonstrate that this is likely to also translate to protein level changes in AD. That RCAN1 was also altered in DLB may speak to converging pathways in DLB and AD. Studies such as Lippa et al. [29] have also shown the presence of Lewy bodies in many Down syndrome brains with AD. Further studies are required to investigate the relevance of RCAN1 in AD, and to also interrogate the potential role/interaction of RCAN1 in the context of neurological diseases such as DLB. We did not observe any differences in RCAN1 levels in WBCs of AD and healthy controls, which may suggest that changes in the level of RCAN1 in the post-mortem brain might be locally/differently regulated compared with peripheral RCAN1 levels. As with the post-mortem brain studies, further work is required to understand how changes in RCAN1 levels in the AD brain may contribute to the disease evolution and progression. Although our results did not show a significant change in the levels of ITSN1 in the AD brain or the other types of dementia, statistical power analysis revealed that if the number of samples was increased to 35 or more per group, the result might reach statistical significance (this would also potentially help overcome issues around varying PMI times and the limited “snapshot” that comes from analysing individuals from an isolated age range). That our current data are in conflict with an existing report suggesting that ITSN1 levels are elevated in AD, this previous study assessed RNA levels only, whereas we assessed protein levels in the current body of work. As such, it is possible that there is disconnect between the RNA and protein regulation/levels for ITSN1, or perhaps there was a cohort-specific difference between studies that would account for this difference. One consideration is the PMI, which could impact protein measurements. A few minutes after death, autolysis occurs resulting in the release of water and enzymes which degrade proteins, carbohydrates and lipids [30]. Environmental factors such as temperature and humidity would also affect the rate of decomposition. For this reason, it is hard to predict how the PMI by itself has affected the protein content of the samples [16]. Similarly, it is not possible to predict in what way our data for levels of ITSN1 protein in dementia brains would have been affected. Nevertheless, the samples with the longer PMI times would likely have had more protein decomposition than the samples with the shorter PMI, which could have affected the level of ITSN1 measured in the samples (whilst there were no significant differences in the PMI between cohorts in the current study, there was a trend for the healthy control group to have shorter PMI times than all other groups). Obtaining better controlled autopsy samples would help mitigate some of these issues. Another issue to consider is whether the presence of significant systemic disease in our sample population might have influenced the outcomes of the current studies, as there were individuals that had sepsis and renal failure amongst other comorbidities [31]. Given the reported role of ITSN1 in a breadth of cellular pathways, then this remains an important caveat of our work.

## Conclusion

There is a potential point of intersection between the neuropathology/disease pathogenesis present in DS and other neurological diseases such as AD and DLB that centers around the regulation of RCAN1 and potentially the associated endocytic pathways. Further investigation is required to understand the relevance of this protein and pathways to disease.

## Figures and Tables

**Table 1:** Demographics for the post-mortem tissues used in this study.

Case No.	Age	Sex	Tissue available	Post- mortem	Diagnosis	Cause of death
				interval (h)		
40/04	91	F	FCX/TCX	3.8	Control	Unknown
19/13	87	F	FCX/ TCX	3.83	Control	Respiratory failure
34/11	91	F	FCX/ TCX	3.33	Control	Unknown
15/08	82	M	FCX/ TCX	13.5	Control	Unknown
41/08	91	F	FCX/ TCX	4.82	Control	Pneumonia
30/04	90	F	FCX/ TCX	3.8	Control	Lung cancer
05/317	73	M	-/ TCX	49	Control	Ischemic Heart Disease, Coronary Artery Atherosclerosis
26/05	91	M	FCX/-	4	Control	Pneumonia
04/104	61	F	FCX/-	71	Control	Acute myocardial infarction, Ischaemic heart disease, Hypertension
05/975	51	M	FCX/-	64	Control	Asthma in a man with cardiac sarcoid
03/480	70	M	-/TCX	13.5	AD	Malignant melanoma, Alzheimer’s Disease
03/653	60	M	FCX/TCX	64.5	AD	Acute upper airways obstruction, Impaction of large food bolus, Dementia (from clinical history)
03/213	80	M	-/ TCX	24	AD	Not available
04/323	82	M	-/ TCX	25	AD	Heart Attack, Arteriosclerosis, Cerebral Atrophy, Dementia
04/013	81	M	-/ TCX	23.5	AD	Unknown
04/164	76	F	-/ TCX	70	AD	Myocardial infarction, Coronary atherosclerosis, Hypertension, Alzheimer’s Disease
04/206	84	M	-/ TCX	73	AD	Faecal Peritonitis, Diverticulitis Carcinoma of Rectum, Abdominal Aortic Aneurysm
03/711	91	F	-/ TCX	34	AD	Malignant mesothelioma
24/11	86	M	-/ TCX	2.92	AD	AD
04/076	83	M	-/ TCX	10	AD	Congestive Cardiac Failure, Generalised Atherosclerosis
04/042	78	F	-/ TCX	19.5	AD	Unknown
03/512	65	M	-/ TCX	56.5	AD	Acute myocardial infarction, Coronary artery atherosclerosis, Dilated cardiomyopathy
04/497	61	M	-/ TCX	13.5	AD	Unknown
04/410	71	M	-/ TCX	66	AD	Urinary sepsis, Dementia
05/840	68	M	-/ TCX	15	AD	Alzheimer’s disease
04/618	83	M	-/ TCX	14	AD	Ischemic heart disease
05/271	83	F	-/ TCX	11.5	AD	Old age, Alzheimer’s disease
05/1055	87	M	-/ TCX	29.5	AD	Ischaemic Heart Disease, Coronary Atherosclerosis
05/684	88		FCX/ TCX	58	AD	Ruptured abdominal aortic aneurysm, Atherosclerosis, Coronary artery, atherosclerosis
05/314	89		FCX/ TCX	43.5	AD	Alzheimer’s disease , Cancer of bowel 2003
04/524	84		FCX/ TCX	28.5	AD	Hypostatic Pneumonia complicating recovery following operative repair of a fractured neck of femur sustained in a fall
05/859	72		-/ TCX	38	AD	Pneumonia −1 week, Dementia of uncertain aetiology - 15 years
05/546	74		FCX/-	61.5	Non-AD	Coronary Artery Atherosclerosis
04/556	75		FCX/-	44	Non-AD	Acute on chronic COAD complicating recovery post op # NOF
05/751	75		FCX/-	11.5	Non-AD	Heart attack
05/1117	77		FCX/-	5	Non-AD	Pneumonia, Dementia - microvascular ischemia and Alzheimer’s disease
05/318	71		FCX/-	25	Non-AD	Pulmonary Embolism, Deep Vein Thrombosis, Myocardial Sarcoid, Acute Enterocolitis
05/728	83		FCX/-	16	Non-AD	Bronchopneumonia, Fractured neck of femur, Dementia, recurrent delirium
03/923	91	M	FCX/-	48	Non-AD	Complications of surgical correction of fractured neck of femur, General debility, Dementia, Hepatic Abscess, IHD, CRF
05/665	77	M	FCX/-	31.5	Non-AD	Respiratory Failure, Emphysema, Lung Cancer, COAD, Diabetes, Ischaemic Heart Disease, Hypertension
03/983	85	M	-/TCX	55	Non-AD	Ischaemic heart disease, Coronary artery atherosclerosis
04/034	73	F	-/TCX	26.5	Non-AD	Pulmonary embolism, Deep Vein Thrombosis, Diverticular disease
04/112	73	M	-/ TCX	22	Non-AD	Ischaemic Heart Disease, Coronary Artery Atherosclerosis, Aortic Stenosis
04/424	75	F	-/ TCX	22.5	Non-AD	Lobar Pneumonia, Coronary Artery Atherosclerosis
04/250	79	M	-/ TCX	31.5	Non-AD	Metastatic malignant pleural mesothelioma
03/964	82	M	-/ TCX	50	Non-AD	Unknown
04/041	82	M	-/ TCX	22	Non-AD	Myocardial infarction
05/898	85	M	-/ TCX	9	Non-AD	Cardiac failure, Hypertension, Heart disease
03/514	59	M	-/ TCX	14.5	Non-AD	Progressive supranuclear palsy, Aspiration pneumonia
05/665	77	M	-/ TCX	31.5	Non-AD	Respiratory Failure, Emphysema, Lung Cancer, COAD, Diabetes, Ischaemic Heart Disease, Hypertension
05/728	83	F	-/ TCX	16	Non-AD	Bronchopneumonia, Fractured neck of femur, Dementia, recurrent delirium
04/249	91	F	FCX/	40.5	DLB	Pneumonia
05/623	87	F	FCX/-	30	DLB	Cardiac arrest - sudden, Ischaemic heart disease –years, Lewy body dementia - many years
04/171	82	F	FCX/-	54	DLB	Unknown
04/245	79	M	-/ TCX	20	DLB	Acute renal failure, Senile debility, Dementia with Lewy bodies
04/157	91	F	-/ TCX	71.5	DLB	Pulmonary Thromboembolism, Thrombosis of left calf
04/510	73	M	-/ TCX	65	DLB	Unknown
05/751	75	M	-/ TCX	11.5	DLB	Heart attack

AD: Alzheimer’s Disease; Non-AD: Non-Alzheimer’s Disease; DLB: Dementia with Lewy Bodies. FCX: Frontal Cortex, TCX; Temporal Cortex.

**Table 2:** Correlation between proteins and metals in AD white blood cells.

	Metal level	RCAN1	ITSN1-S	ITSN1-L
**AD Iron (μg/g)**	398.3 ± 42.2, n=48	r=0.09 (−.027 to 0.37)	r=−0.18 (−0.48 to 0.15)	r=−0.11 (−0.43 to 0.24)
**HC Iron (μg/g)**	414.8 ± 88.4, n=17	r=−0.38 (−0.74 to 0.14)	r=−0.23 (−0.73 to 0.43)	r=−0.28 (−0.75 to 0.38)
**AD Zinc (μg/g)**	176.3 ± 12.1, n=49	r=0.23 (−0.06 to 0.48)	r=0.12 (−0.21 to 0.43)	r=−0.79 (−0.40 to 0.26)
**HC Zinc (μg/g)**	171.3 ± 21.9, n=19	r=−0.28 (−0.66 to 0.21)	r=−0.22 (−0.72 to 0.44)	r=−0.24 (−0.73 to 0.42)
**AD Copper (μg/g)**	9.6 ± 0.8, n=47	r=0.22 (−0.08 to 0.48)	r=0.25 (−0.09 to 0.53)	r=0.13 (−0.21 to 0.45)
**HC Copper (μg/g)**	9.9 ± 1.6, n=19	r=−0.17 (−0.59 to 0.32)	r=−0.22 (−0.73 to 0.43)	r=−0.17 (−0.69 to 0.48)
**AD Calcium (μg/g)**	3752 ± 265.3, n=47	r=0.11 (−0.19 to 0.39)	r=0.18 (−0.16 to 0.47)	r=0.07 (−0.28 to 0.41)
**HC Calcium (μg/g)**	3349 ± 323.2, n=18	r=−0.37 (−0.71 to 0.11)	r=−0.21 (−0.72 to 0.45)	r=−0.24 (−0.73 to 0.41)
**AD Magnesium (μg/g)**	2311 ± 125.8, n=46	r=0.16 (−0.15 to 0.43)	r=0.06 (−0.28 to 0.39)	r=−0.18 (−0.48 to 0.16)
**HC Magnesium (μg/g)**	2137 ± 208.5, n=18	r=−0.37 (−0.71 to 012)	r=−0.30 (−0.76 to 0.36)	r=−0.35 (−0.79 to 0.31)
**AD Manganese (μg/g)**	1.1 ± 0.1, n=49	r=0.16 (−0.13 to 0.43)	r=0.11 (−0.23 to 0.42)	r=−0.12 (0.44 to 0.23)
**HC Manganese (μg/g)**	1.1 ± 0.2, n=19	r=−0.35 (−0.7 to 0.14)	r=−0.16 (−0.69 to 0.48)	r=−0.21 (−0.72 to 0.44)
**AD Selenium (μg/g)**	2.5 ± 0.2, n=45	r=0.005 (−0.29 to 0.30)	r=0.07 (−0.28 to 0.40)	r=0.05 (−0.32 to 0.40)
**HC Selenium (μg/g)**	2.8 ± 0.3, n=20	r=0.13 (−0.36 to 0.56)	r=−0.18 (−0.71 to 0.47)	r=−0.19 (−0.71 to 0.46)
**AD Aluminium (μg/g)**	539.4 ± 47.9, n=46	r=0.08 (−0.22 to 0.37)	r=0.07 (−0.28 to 0.4)	r=−0.13 (−0.46 to 0.22)
**HC Aluminium (μg/g)**	460.3 ± 68.5, n=18	r=−0.23 (−0.64 to 0.28)	r=−0.25 (−0.74 to 0.41)	r=−0.31 (−0.77 to 0.35)
**AD Lead (μg/g)**	2.2 ± 0.2, n=46	r=0.17 (−0.13 to 0.45)	r=0.292 (−0.051 to 0.57)	r=0,55 (0.24 to 0.76)
**HC Lead (μg/g)**	2.9 ± 0.6, n=19	r=−0.056 (−0.51 to 0.42)	r=−0.07 (−0.64 to 0.55)	r=−0.22 (−0.77 to 0.52)

This table shows the level of metals in the white blood cells (μg/g), and Pearson correlation coefficient (r value and 95% confidence interval in the bracket) expressing the correlations between proteins (RCAN1, ITSN1-S and ITSN1-L) and metals in Alzheimer’s disease (AD) white blood cells compared with the healthy controls (HC). There are no correlation between any of the proteins and metals. The level of different metals in the WBC was the same between the groups of AD and control samples. A two-tailed Pearson’s correlation test was used for statistical analysis.
